# Fabrication of a biological metal–organic framework based superhydrophobic textile fabric for efficient oil/water separation

**DOI:** 10.1038/s41598-022-19816-y

**Published:** 2022-09-15

**Authors:** M. E. Mohamed, B. A. Abd-El-Nabey

**Affiliations:** Chemistry Department, Faculty of Science, Alexandria University, PO Box 426, Alexandria, 21321 Egypt

**Keywords:** Chemical engineering, Nanoscale materials

## Abstract

In response to the industry's difficulty in properly separating oily wastewater discharge, researchers are investigating enhanced oil/water separation materials. In this work, a cost-effective and environmentally friendly superhydrophobic textile fabric was fabricated for effective oil–water mixture and emulsion separation. A biological metal–organic framework consisting of copper as a core metal and aspartic acid as a linker (Cu-Asp MOF) was used to improve the surface roughness of the pristine textile fabric, and stearic acid was used to lower its surface energy. The thermal gravimetric analysis investigated the prepared Cu-Asp MOF's thermal stability. X-ray spectroscopy and Fourier-transform infrared spectroscopy studied the crystal orientation and chemical composition of the Cu-Asp MOF, Cu-Asp MOF@SA, pristine textile fabric, and superhydrophobic textile fabric, respectively. The surface morphology of the pristine and modified textile fabric was studied by scanning electron microscope. The wettability results showed that the prepared superhydrophobic textile fabric has a water contact angle of 158° ± 1.3 and water sliding angle of 2° ± 0.2°. The prepared superhydrophobic textile fabric showed excellent oil–water mixture and emulsion separation performance, oil absorption capacity, chemical stability, mechanical abrasion resistance, and a high flux rate. These outstanding characteristics of the prepared superhydrophobic textile fabric greatly increase the possibility for practical applications.

## Introduction

Oil pollution issues such as accidental crude oil spills and the discharge of oily wastewater due to industrial activities have gotten much attention and spurred a debate regarding oil/water separation^1–8^. Many technologies such as centrifugation separation^9^, oil skimmers^10^, demulsifiers^11^, microbiological degradation^12^, gravity separation^13^, vacuum dehydration^14^, and functional material absorption^15^ have been developed for separating oil from oily wastewater. Researchers have been developing more effective separation methods for oil/water separation to address the limitations of present systems, such as large equipment, high energy consumption, complex separation process, easy creation of secondary pollutants, and low separation efficiency^16–18^. Because of its ease of separation equipment and low energy consumption, membrane separation techniques such as metal mesh^16,19^, fabric^20,21^, sponge^22,23^, and paper^23,24^ are the most efficient techniques for separating oil–water combinations.

Because of their differing inherent wettability of oil and water, superhydrophobic membranes are currently thought to be promising in oil–water separation^2,25–30^. Different superhydrophobic films with exceptional water repellency can be achieved by increasing the roughness of the surface, the first characteristic necessary to attain superhydrophobicity, and lowering surface energy, the second characteristic necessary to attain superhydrophobicity^31–35^. To improve roughness, researchers used a variety of nanomaterials, including SiO_2_^3^, TiO_2_^22^, ZnO^36^, CuO^37^, carbon nanotubes^38^, and metal–organic frameworks^39^. Immersion^40,41^, electrodeposition^42^, electrospinning^43^, plasma etching^44^, layer self-assembly^45^, electrochemical anodic oxidation^46^, chemical vapour deposition^47^, phase separation^48^, dipping^49^, spraying^50^, and sol–gel methods^51^ have all been proposed for the preparation of superhydrophobic coatings.

Metal–organic frameworks (MOFs) based membranes with great wettability can be used to enable selective and effective separation of oil–water mixtures^39,52^. MOFs are an innovative mixture of natural porous materials with a periodic network structure generated by core metal ions and organic linkers^53^. MOFs have gotten a lot of press because of their unique properties. They have a complex and varied framework structure, ordered and regular pore structure, a high specific surface area, and the capacity to move functional groups in pores and surfaces^53,54^. MOFs are used in many applications, including bioreactors^55^, proton conductive materials^53^, medication delivery^56^, chemical sensors, water purification^57^, catalysis^58^, and gas separation and storage^59^. MOFs are manufactured by microwave-assisted synthesis^60^, colloidal deposition method^61^, layer-by-layer synthesis^62^, evaporation induced crystallization^63^, and solvothermal/ hydrothermal synthesis^64^. Complex procedures, high energy consumption, the need for modern equipment, long reaction times, difficult process regulation, and the use of undesired anions through utilizing metal salts are all drawbacks of these approaches. Compared to traditional methods, electrochemical methods provide a variety of advantages, including mild reaction conditions, convenience of use, and clean-up^65^. Biological metal–organic frameworks, Bio–MOFs, have lately gained popularity as green, biodegradable, renewable framework material^66^. Bio–MOFs use nucleobases, peptides, amino acids, and saccharides as building blocks^66^.

This research aims to construct a Bio-MOF-based superhydrophobic coating on textile fabric for oil/water separation. A Bio-MOF consists of copper as a core metal and aspartic acid as a linker, Cu-Asp MOF, which was manufactured using an electrochemical method. The prepared Cu-Asp MOF was immersed in an ethanolic solution of stearic acid. The Cu-Asp MOF modified with stearic acid, Cu-Asp MOF@SA, was sprayed on the textile fabric to produce the superhydrophobic coating. The composition, crystal orientation, and thermal stability of the prepared Cu-Asp MOF were investigated. The prepared superhydrophobic textile fabric's surface composition, morphology, wettability, oil–water mixture and emulsion separation performance, oil absorption capacity, Flux, mechanical abrasion resistance, and chemical stability were studied.

## Experimental

### Materials

Stearic acid, aspartic acid, methyl red, silicone oil (viscosity 500 cSt at 20 °C), petroleum ether, n-hexane, sulphuric acid (99%), Tween 80 and sodium hydroxide were gained from SIGMA ALDRICH. Pure textile fabric and a sheet of copper metal were purchased from a local store.

### The fabrication of Cu-Asp MOF

A copper sheet (50 mm × 20 mm × 1.0 mm) was polished with sandpaper (300 and 800 grit) to erase the oxide layer at the surface of copper and rinsed with distilled water and ethanol. A solution of 100 mL aspartic acid (4.5 gL^−1^) was served as the electrochemical bath. A direct current was used to start the electrochemical synthesis, with two polished copper sheets serving as anode and cathode. The current density was adjusted to 0.05 A/cm^2^ for one hour at a temperature of 55 ± 1 °C. A blue precipitate formed at the anode as the process continued. The formed precipitate was rinsed with ethanol and dried at room temperature; then, it was scratched. The obtained MOF was dried in an oven at 100 °C for two hours. The formed copper aspartic acid MOF was designated as Cu-Asp MOF.

### Superhydrophobic textile fabric fabrication

**Figure 1:**
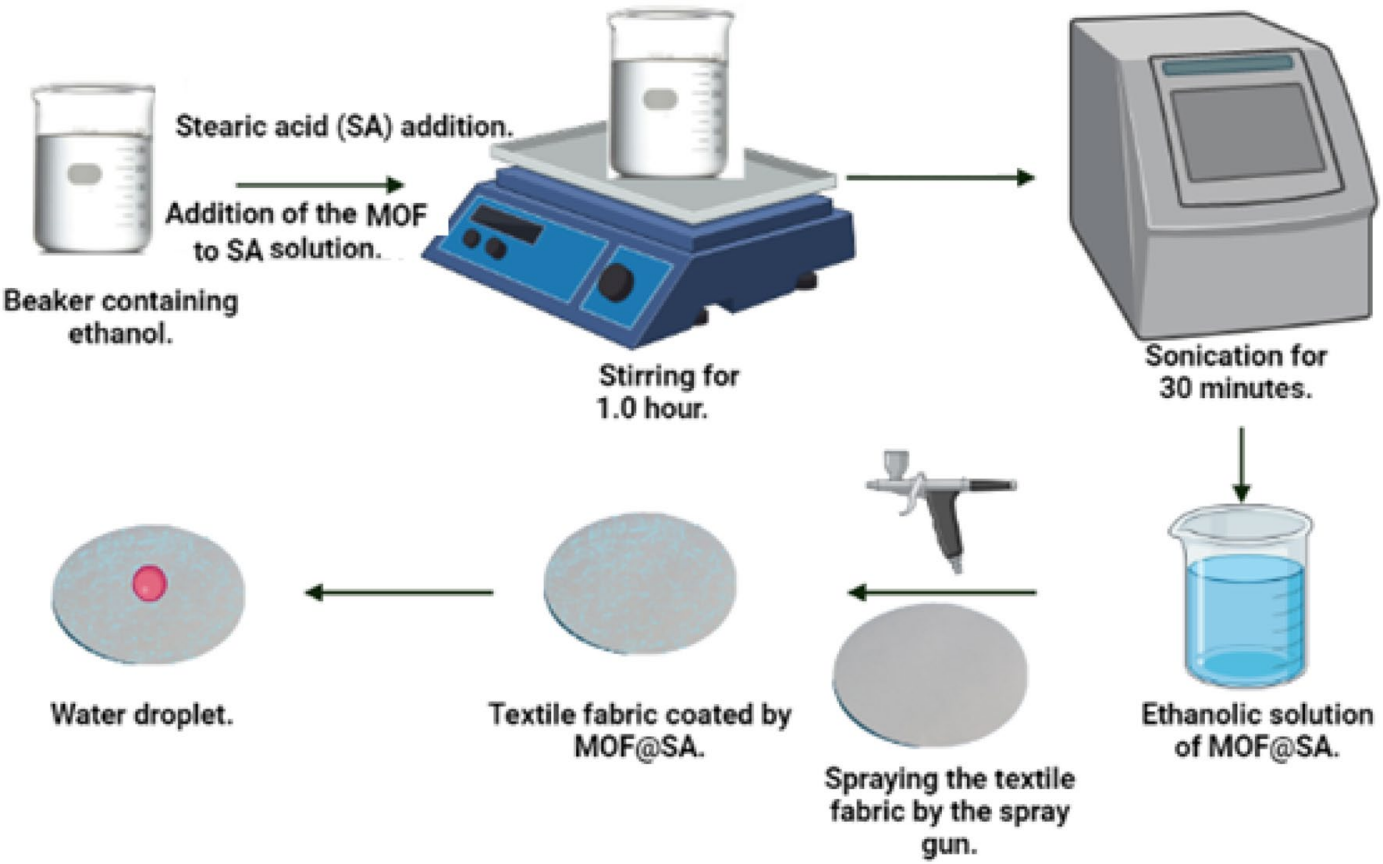
Schematic diagram of the manufacturing of superhydrophobic textile fabric, TF@Cu-Asp MOF@SA.

An ethanolic solution of 250 mL of Cu-Asp MOF (0.8 gL^−1^) and stearic acid (8.0 gL^−1^) was stirred for one hour, then sonicated for half hour, and a solution of Cu-Asp MOF@SA was prepared**.** A Circular textile fabric (diameter of 10.5 mm) was sprayed with Cu-Asp MOF@SA. The modified textile fabric, TF@ Cu-Asp MOF@SA, was then rinsed with ethanol and dried for two hours at 60 °C. Figure 1 shows the fabrication scheme for superhydrophobic textile fabric, TF@Cu-Asp MOF@SA.

### Characterization

A scanning electron microscope, SEM, was used to examine the textile fabric morphology before and after modification (model JSM-200 IT, JEOL). An energy dispersive x-ray spectrometer was used to determine the elemental composition of the prepared materials and surfaces (EDX JEM-2100 Japan). Fourier-transform infrared spectroscopy, FTIR, was used to investigate the chemical composition of the surface (model: Bruker Tensor 37 FTIR). The FTIR spectra were taken in the 4000–400 cm^−1^ range. Furthermore, an X-ray diffractometer (Bruker D2 phaser) was used to distinguish the crystal phase and crystallinity. Thermogravimetric analysis was used to determine the Cu-Asp MOF's thermal stability (TGA-Shimadzu-50). An optical contact angle goniometer (Rame-hart CA instrument, model 190-F2) was used to measure the water contact angle (WCA) and sliding angle (WSA) with water droplets of 5 µl. The contact angle values are the averages of four measurements taken at various locations on the surface of the manufactured superhydrophobic textile fabric. Sodium hydroxide and sulfuric acid were used to alter the water droplets' pH. An abrasion test measured the mechanical stability of the prepared superhydrophobic textile fabric. The prepared superhydrophobic textile fabric was placed on 600 mesh sandpaper, and a weight of 100 g was applied to it and pulled for 10 cm, named one abrasion cycle. The created superhydrophobic textile fabric's chemical stability was investigated by examining the impact of immersion times of various prepared superhydrophobic samples in solutions of pH ranging from 1 to 13 for 1, 2, 3, 4, 5, and 6 h on the value of WCA^67^. Meantime, water CAs on all samples are measured in three different positions every hour, which is repeated 3 times.

The chemical stability results presented here are the averages of the three tests.

### Absorption capacity measurements

The prepared superhydrophobic textile fabric's absorption capability was determined by immersing a piece of the fabric in silicone oil, petroleum ether, or n-hexane for one minute, draining for a few seconds, and wiping with filter paper to remove the excess organic solvents. Using weights measured at room temperature, the absorption capacity (k) of silicone oil, petroleum ether, and n-hexane was determined by the following equation^68^:

1 $$ {\text{k }} = {\text{ M}}_{{1}} /{\text{M}}_{0} $$

M_0_ represents the weight of the modified superhydrophobic textile fabric sample, whereas M_1_ represents the weight of the sample after organic solvent absorption.

### Measurements of oil/water separation performance

The oily wastewater was made of a 50 mL oil/water mixture with a volume ratio of 1:1. Methyl red was employed to color the distilled water. The oil phases were chosen from various organic solvents such as silicone oil, petroleum ether, and n-hexane. The oil-in-water (O/W) emulsion was formed by mixing up water, n-hexane, and Tween 80 in a mass ratio of 40:9.9:0.1 and then sonicating by ultrasonication at 25 °C for 1.5 h. During oil/water separation, the prepared superhydrophobic textile fabric was used as a filter membrane. When the oily wastewater was put into the separation system, the oil penetrated the modified textile fabric and fell into the lower receipting container, leaving water in the upper container. The following equation was used to compute the separation efficiency (W)^68^:

2 $$ {\text{W }} = \, \left( {{\text{M}}_{{2}} /{\text{M}}_{{3}} } \right) \, \times {1}00 \, \% $$

M_3_ and M_2_ are the starting and collected oil weights, respectively. The total organic carbon (TOC) of the aqueous phase after separation process was measured using total organic carbon analyzer (HACH DR 2700) to confirm the separation efficiency.

The following equation was used to estimate the Flux of the modified textile fabric^68^:

3 $$ {\text{Flux}} = {\text{V}}/\left( {{\text{S}} \times {\text{t}}} \right) $$

where V is the volume of permeating liquid, S is the effective area of the film, and t is the time it takes for the liquid to pass through the film.

## Results and discussion

### Thermogravimetric results of the prepared Cu-Asp MOF

**Figure 2:**
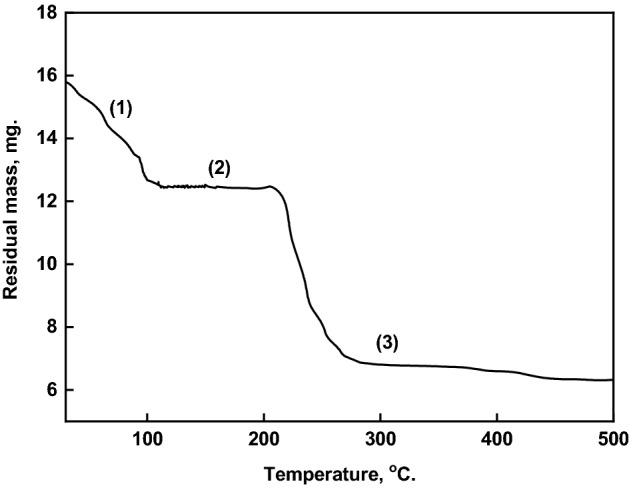
Thermogravimetric analysis in air atmosphere of Cu-Asp MOF.

The thermograms for the prepared Cu-Asp MOF under an air atmosphere are presented in Fig. 2. The thermograms are divided into three regions. The first region is within the temperature range between 30 and 100 °C. In the first region, weight reduction was noticed due to the volatilization of adsorbed water. The second region ranges from 100 to 215 °C. The weight loss observed at 215 °C is attributed to the breakage of the bridge between the metal cluster and the organic linker and the decomposition of Cu-Asp MOF. The third region ranges from 215 to 285 °C. The weight loss observed in the third region is due to the decomposition of the linker, aspartic acid^69^. So, we can conclude that the prepared Cu-Asp MOF is thermally stable until 215 °C.

### XRD results

**Figure 3:**
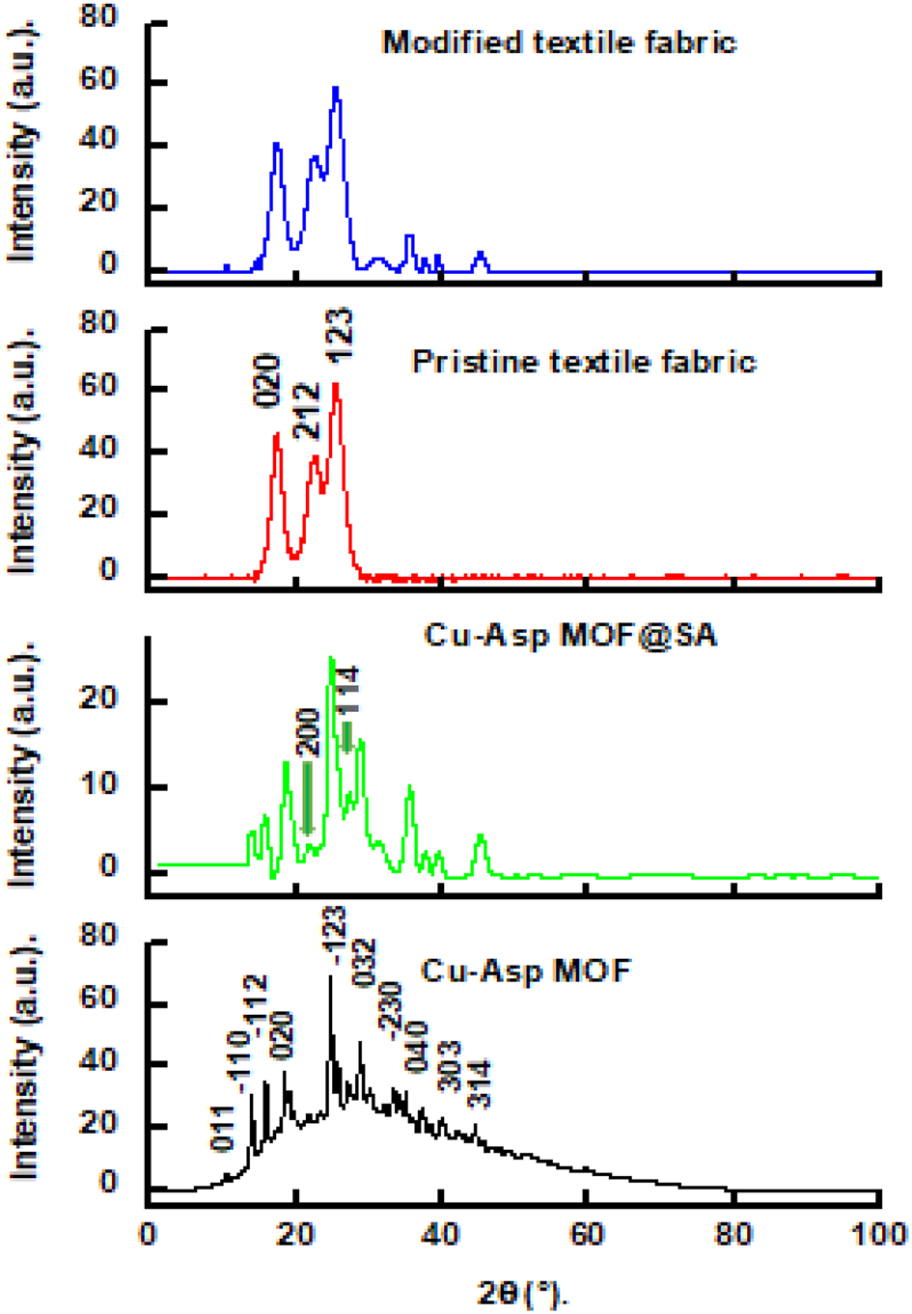
XRD pattern of Cu-Asp MOF, Cu-Asp MOF@SA, pristine textile fabric, and modified textile fabric.

The XRD technique was used to know the composition and the crystal orientation of the Cu-Asp MOF, Cu-Asp MOF@SA, pristine textile fabric, and modified textile fabric. Figure 3 depicts the XRD patterns of the Cu-Asp MOF, Cu-Asp MOF@SA, pristine textile fabric, and modified textile fabric. The prepared Cu-Asp MOF XRD pattern implies that the deposits contain copper, oxygen, and carbon. The main diffraction peaks are observed at 2Ɵ value equals 10.48°, 14.15°, 16.10°, 18.70°, 24.88°, 29.13°, 35.32°, 37.22°, 39.92° and 44.67°. These peaks are characteristics of the monoclinic crystal system of the prepared Cu-Asp MOF. The Scherrer equation determines the average grain size of the prepared Cu-Asp MOF sample; the crystallite size equals 19.6 nm. The XRD pattern of prepared Cu-Asp MOF@SA shows the same pattern of Cu-Asp MOF with two extra diffraction peaks at 2Ɵ values equal to 21.79° and 27.44°, indicating that the Cu-Asp MOF was grafted by stearic acid^70^. The XRD pattern of pristine textile fabric shows diffraction peaks at 17.31°, 22.40°, and 25.22° which are lattice faces of alpha polyamide contributed by nylon textile substrate. The modified textile fabric has a similar diffraction peak compared to the pristine textile fabric and Cu-Asp MOF@SA, indicating that Cu-Asp MOF was successfully grafted on the textile fabric. Interestingly, all peak intensities of modified textile were decreased, which attributed to the grafting of Cu-Asp MOF at the textile fabric would shield the signal of polyamide of nylon textile fabric^71–75^.

### FTIR results

**Figure 4:**
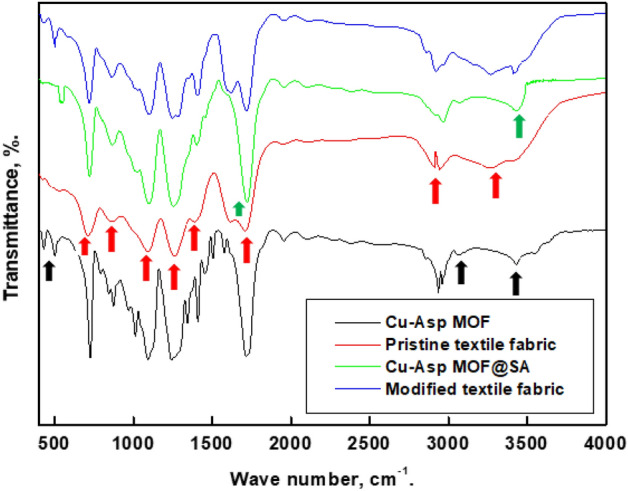
FTIR of the Cu-Asp MOF, Cu-Asp MOF@SA, pristine and modified textile fabric.

FTIR spectroscopy techniques investigated the chemical composition of the Cu-Asp MOF, Cu-Asp MOF@SA, pristine textile fabric, and modified textile fabric, Fig. 4. The spectrum of pristine textile fabric displays a peak at 3430 cm^−1^ attributed to the N–H stretch of secondary amide and the two peaks at 2912 cm^−1^ and 2965 cm^−1^, assigned to the -CH_2_- asymmetry and symmetry vibration^76^. The peak at 1715 cm^−1^ is attributed to the C=O stretch, and the peak at 1409 cm^−1^ is caused by the bending vibration of C-H. While the peaks at 1093 and 1242 cm^−1^ are attributed to C-N stretch, the peak at 873 cm^−1^ is due to C–H bending, and the peak at 726 cm^−1^ is due to N–H out of plane bending. The spectrum Cu-Asp MOF displays the same peaks as that of the pristine textile fabric except that the appearance of two peaks at 3269 and 3433 cm^−1^ attributed to N-H_2_ stretch of primary amine of aspartic acid^74,77–80^. The two peaks at 501, and 431 cm^−1^ attributed to the Cu−O and Cu−N bonds vibration, respectively^81–85^. This result confirms that the copper is coordinated with both the nitrogen and oxygen of aspartic acid. The spectrum Cu-Asp MOF@SA displays the same peaks as that of the Cu-Asp MOF with a shift in C=O stretch peak and N-H_2_ stretch peak located at 1719 cm^−1^ and 3444 cm^−1^, indicating the grafting of Cu-Asp MOF with stearic acid. The spectrum of modified textile fabric displays the same peaks as Cu-Asp MOF@SA, indicating that the prepared Cu-Asp MOF@SA grafted the pristine textile fabric.

### Energy dispersive x-ray spectrometer (EDX) results

**Figure 5:**
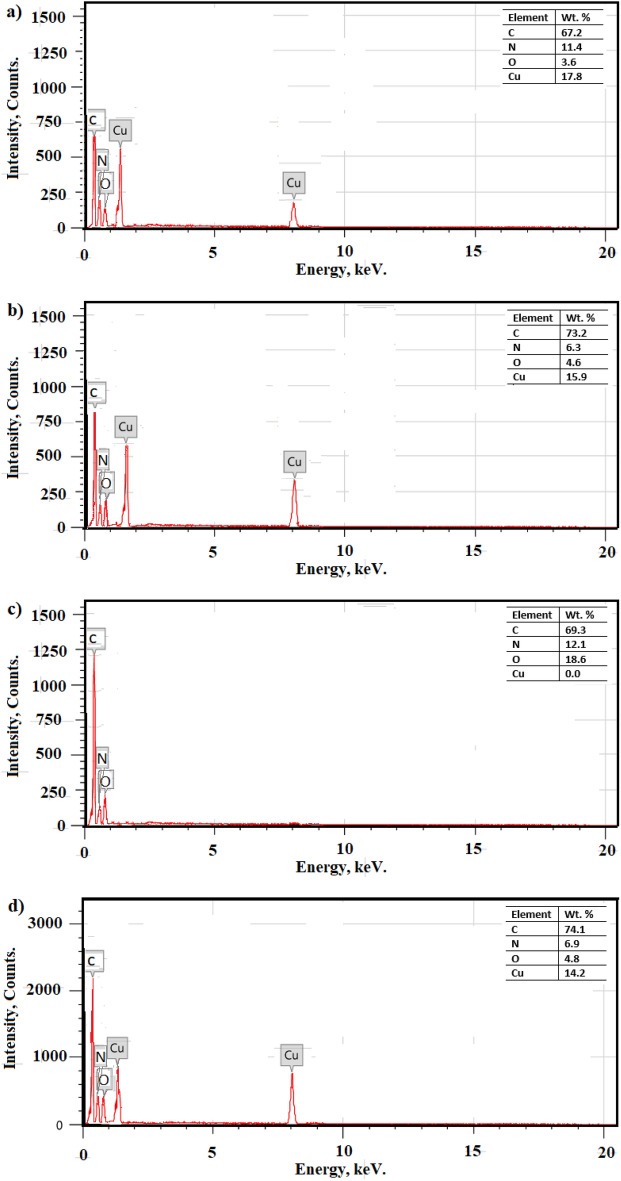
EDX micrographs of the (**a**) Cu-Asp MOF, (**b**) Cu-Asp MOF@SA, (**c**) pristine textile fabric, and (**d**) modified textile fabric.

The EDX was used to investigate the chemical composition of the Cu-Asp MOF, Cu-Asp MOF@SA, pristine textile fabric, and modified textile fabric, Fig. 5. The micrograph of Cu-Asp MOF shows peaks of carbon, oxygen, nitrogen, and copper. The micrograph of Cu-Asp MOF@SA shows the same peaks of Cu-Asp MOF but with a higher weight percent of carbon and oxygen, indicating that the stearic acid is successfully grafted on the prepared MOF. The pristine textile fabric micrograph shows carbon, oxygen, and nitrogen peaks. The modified textile fabric micrograph shows the same pristine peaks with an extra peak of copper, indicating that the prepared Cu-Asp MOF@SA was grafted on the textile fabric.

### SEM and wettability of the pristine and modified textile fabric results

**Figure 6:**
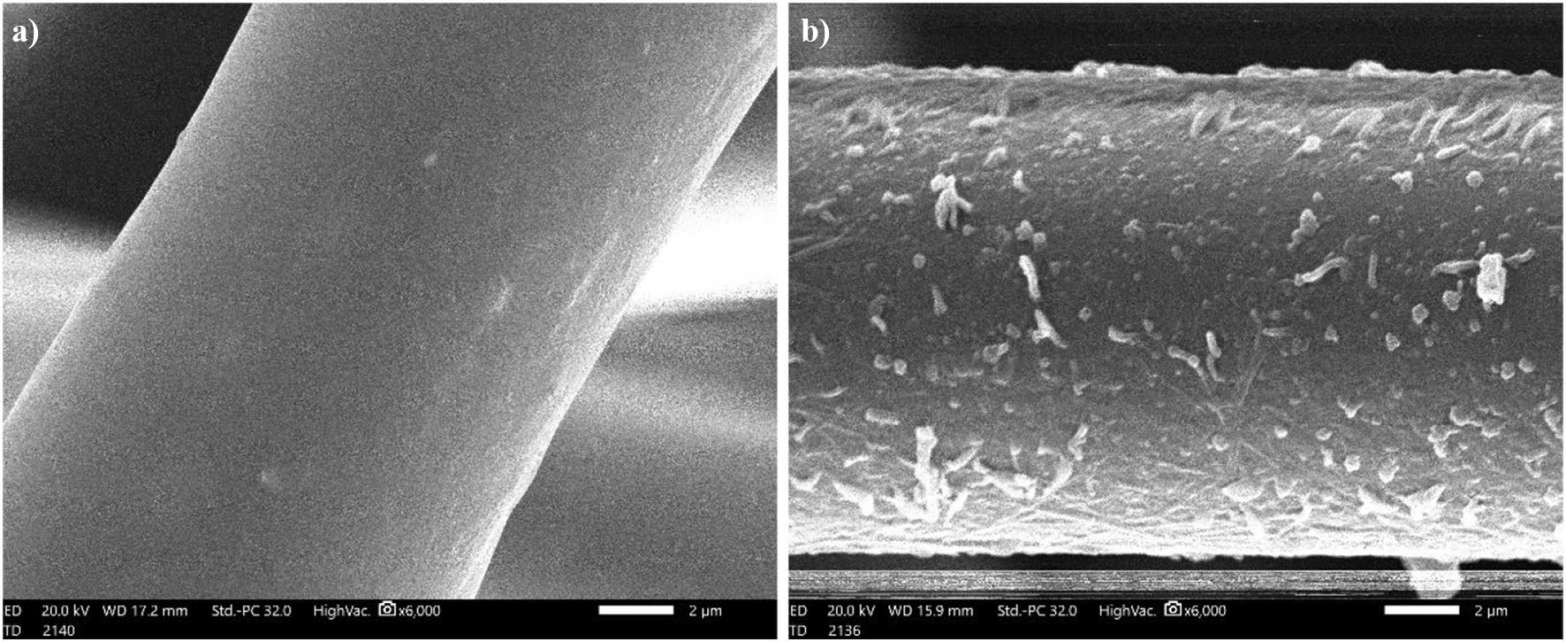
SEM images of the pristine (**a**) and modified textile fabric (**b**).

Surface roughness and surface chemical composition determine the superhydrophobic property. The SEM was used to investigate the surface topography of the pristine and modified textile fabric. The SEM micrographs of pristine and modified textile fabric are shown in Fig. 6. As illustrated in the figure, the fibers of pristine textile have a relatively smooth surface. While numerous micro/nano structures cover the modified textile fabric fibers, the fibers' surface roughness was significantly improved.

**Figure 7:**
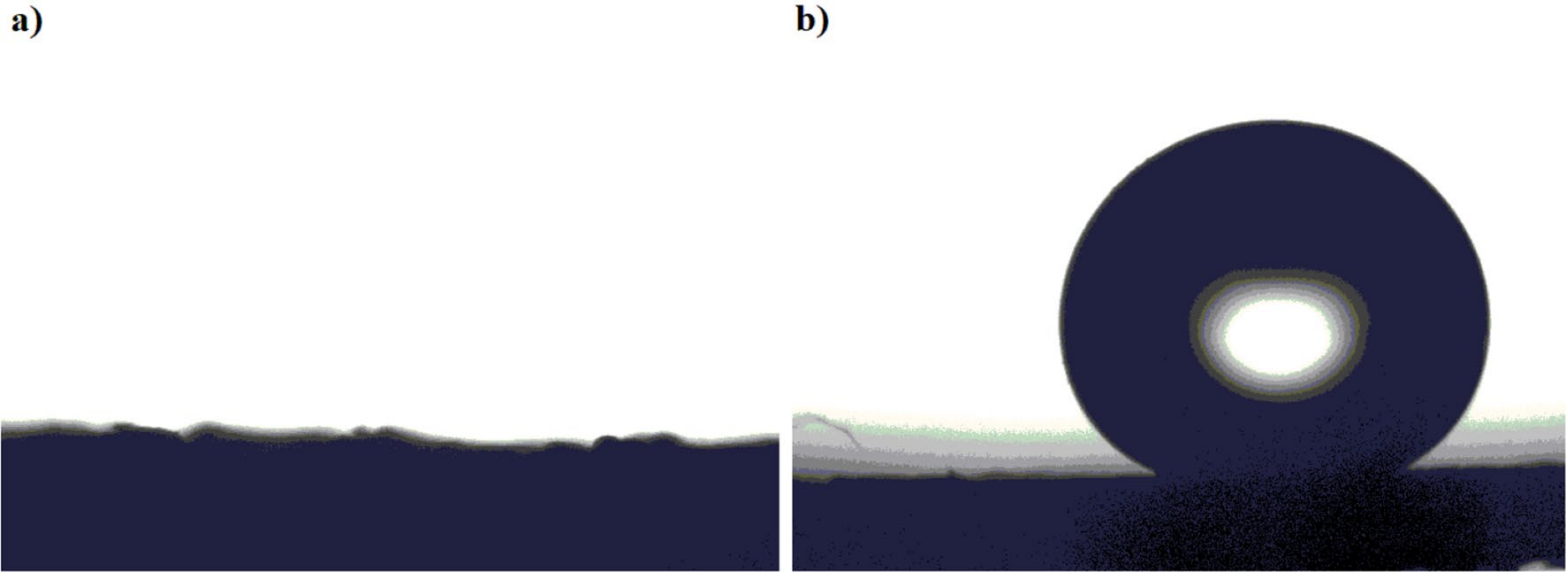
Images of water droplets at the pristine surface (**a**) and modified textile fabric (**b**).

The wettability of the pristine and modified textile fabric was investigated by the measurement of WCA, Fig. 7. As shown in Fig. 7, the surface of the pristine textile fabric is hydrophilic, WCA near 0^◦^, and the water droplets do not slide even if the textile fabric is upset. The modified textile fabric has a water contact angle, WCA, of 158° ± 1.3 ^o^, and a water sliding angle, WSA, of 2° ± 0.2° (see video S1), which showed excellent superhydrophobic properties.

### Absorption capacity measurements

The prepared superhydrophobic textile fabric's absorption capability to n-hexane, petroleum ether, and silicone oil was determined. Although the pristine textile fabric absorbed both water and oil, the developed superhydrophobic modified textile fabric selectively absorbed only oil. The high porosity, low density, superhydrophobic characteristics, and capillary force of the prepared modified textile fabric contribute to its rapid absorption of organic solvents^68^.

**Figure 8:**
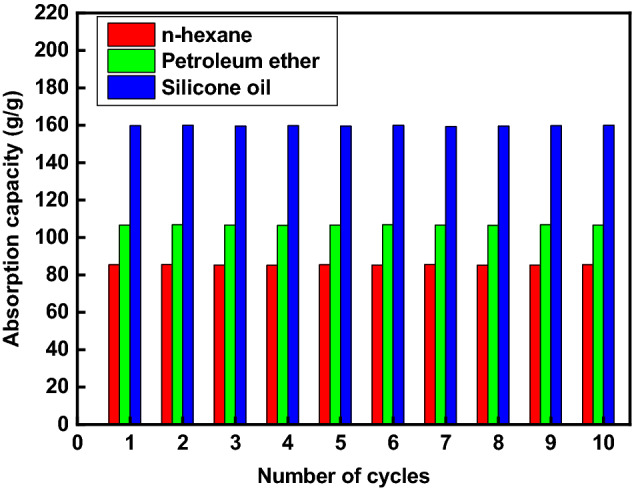
Absorption capacity of various oil/water combinations as a function of cycle count.

The prepared superhydrophobic textile fabric's absorption capacity to n-hexane, petroleum ether, and silicone oil was repeated ten times, Fig. 8. After each cycle, the oil absorption capacity was measured, and the desorption process of the organic solvents was done by squeezing the prepared superhydrophobic textile fabric. The absorption capacity of silicone oil is the highest, while n-hexane is the lowest, as shown in Fig. 8. The developed modified textile fabric has absorption capacities of 85.4, 106.59, and 159.712 g/g for n-hexane, petroleum ether, and silicone oil, respectively. It is reported in many previous works that when the oil density and viscosity increase, the absorption capacity increases^86–92^. This may be explained by the fact that oil with a higher density and viscosity will take longer to release from the superhydrophobic membrane, leaving more oil in the porous structure and increasing its absorption capacity^89^. In comparison to the light oil, the higher density oil will weigh more in the same pore volume of the superhydrophobic membrane so that the higher density oil will have a higher absorption capacity.

This result could be explained by the density difference between these organic solvents; the solvent with a higher density has a greater absorption capacity. Even after ten cycles, the absorption capacity remains practically constant, showing that the developed superhydrophobic textile fabric for oil separation is recyclable. The contact angle of the prepared superhydrophobic/oleophilic textile fabric was 154° ± 1.4° after the ten cycles reflecting its high mechanical stability. So, the modified textile fabric maintains its superhydrophobic characteristics even after ten absorption processes to the organic solvents under study.

Furthermore, these absorption capabilities (wt/wt) are greater than those of previously reported absorbents such as textile fabric grafted by CuS nanoparticles and silicone elastomer polydimethylsiloxane (71.43–156.24)^93^. Textile fabric modified with phytic acid and polydimethylsiloxane (71.43–156.24)^94^. The developed modified textile fabric's high absorption capacity effectively absorbs spilled oils and other organic liquids.

### Oil/water separation efficiency

**Figure 9:**
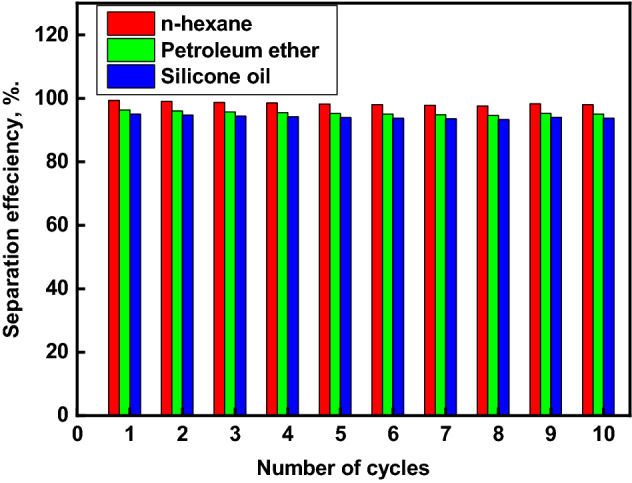
Separation efficiency of the modified textile fabric for different oil/water mixtures with the number of separation cycles.

Figure 9 shows the results of 10 cycles of separation efficiency of the manufactured superhydrophobic textile fabric with various oil/water mixes. The modified textile fabric has a good separation efficiency for the various oil/water mixes under investigation. The separation efficiency for n-hexane/water mixtures, petroleum ether/water mixtures, and silicone oil/water mixtures equal 99.4, 96.4, and 95.0%, respectively (see video S2, which shows the separation process of n-hexane/water mixtures). As a result, the superhydrophobic textile fabric exhibits remarkable oil selectivity. The separation efficiency was confirmed by measuring the TOC of the aqueous phase after separation process. The TOC of the aqueous phase after the separation process of n-hexane/water, petroleum ether/water, and silicone oil/water mixtures are 12 ppm, 14 ppm, and 17 ppm, respectively. The small values of TOC of the aqueous phase after separation process confirm the high separation efficiency of the oil/water mixture using the prepared superhydrophobic textile fabric.

The figure also demonstrates that the first cycle has the highest separation efficiency. The separation efficiency was still greater than 93.5 percent after ten oil/water separation cycles. The minor loss of collected oil during the separation process is primarily due to oil adherence to the funnel and sample. Furthermore, the oil's volatility should contribute to a drop in separation efficiency^68^. Due to its porosity, the oil penetrates through the modified textile fabric, and the rough features make it simple to build oil channels, resulting in a high oil flux. Furthermore, this absorbent's separation effectiveness (%) is greater than that of previously reported absorbents, such as textile modified with polyvinyl alcohol (91–95%)^95^. Textile grafted with fluoroalkyl silane-modified iron oxide nanoparticles in polydimethylsiloxane (92.2–93.5)^96^.

**Figure 10:**
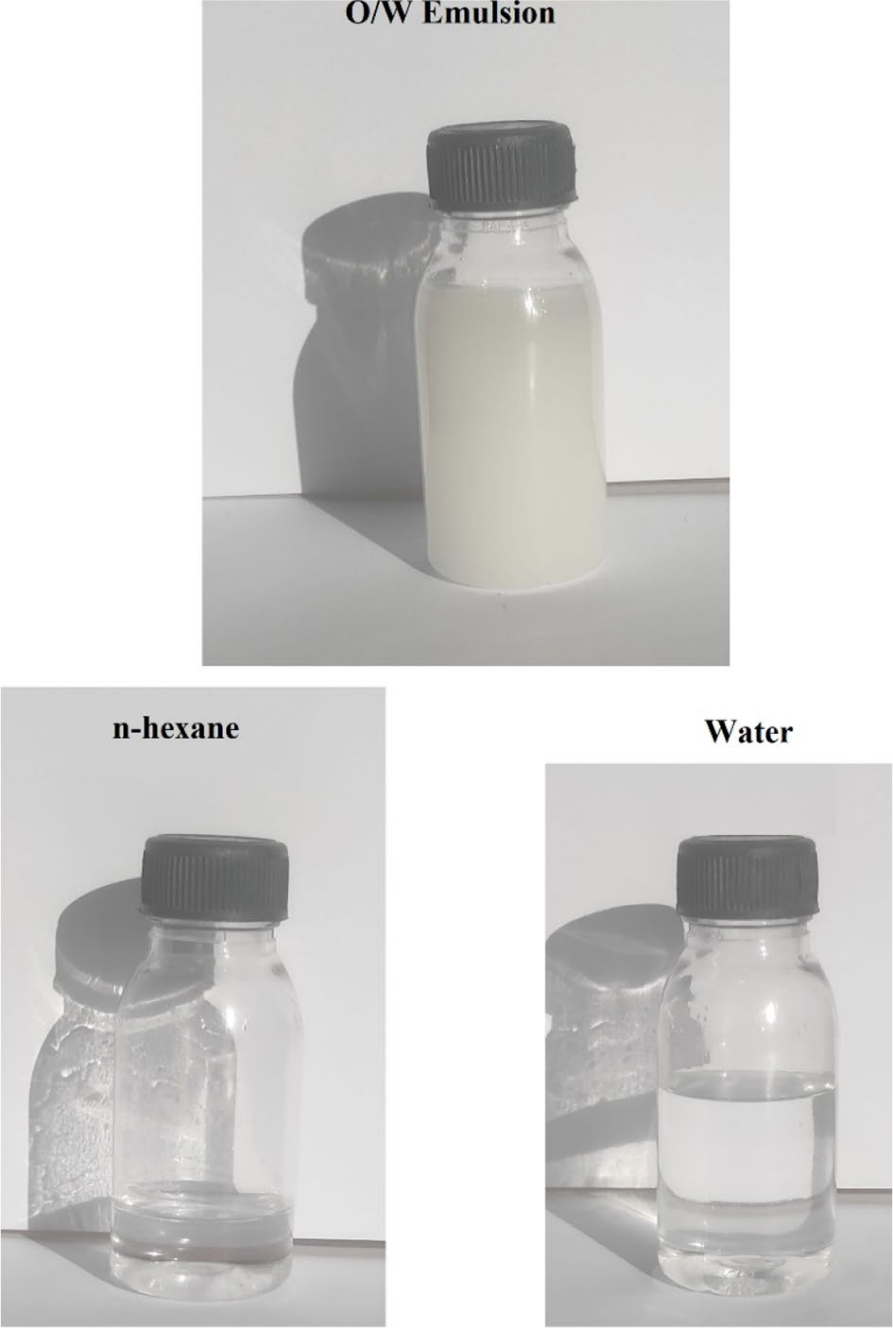
Images of oil-in-water emulsion before and after the separation process.

Furthermore, the same method can be used to separate O/W emulsions that have been stabilized by surfactants. Oil/water emulsion separation is quite challenging, especially when surfactants are present^28^. Here, a Tween 80-stabilized n-hexane in water emulsion was prepared in order to explore the separation of O/W emulsion. The prepared modified textile fabric was used for the O/W emulsion separation. As soon as the emulsion was poured into the top funnel, the oil permeated through the modified fabric and was collected there, leaving the water on the upper funnel (see video S3, which shows the separation process of the oil/water emulsion). Figure 10 shows the emulsion images before and after the separation process. It is obvious that the colour is transformed from milky to clear after one separation cycle, and no n-hexane droplets were observed in the water phase. The separation efficiency for Tween 80-stabilized n-hexane in water emulsion equals 90.1% which is calculated using Eq. 2. The separation efficiency of the O/W emulsions was confirmed by measuring the TOC of the aqueous phase after separation process. The TOC of the aqueous phase after separation is 22 ppm. The TOC values of the water after filtration are smaller than many previously reported values indicating high separation efficiency of the prepared superhydrophobic membrane toward O/W mixture and emulsion^97–99^. These results show that the prepared superhydrophobic textile fabric can be used for O/W mixture and emulsion separation.

### Mechanical stability

**Figure 11:**
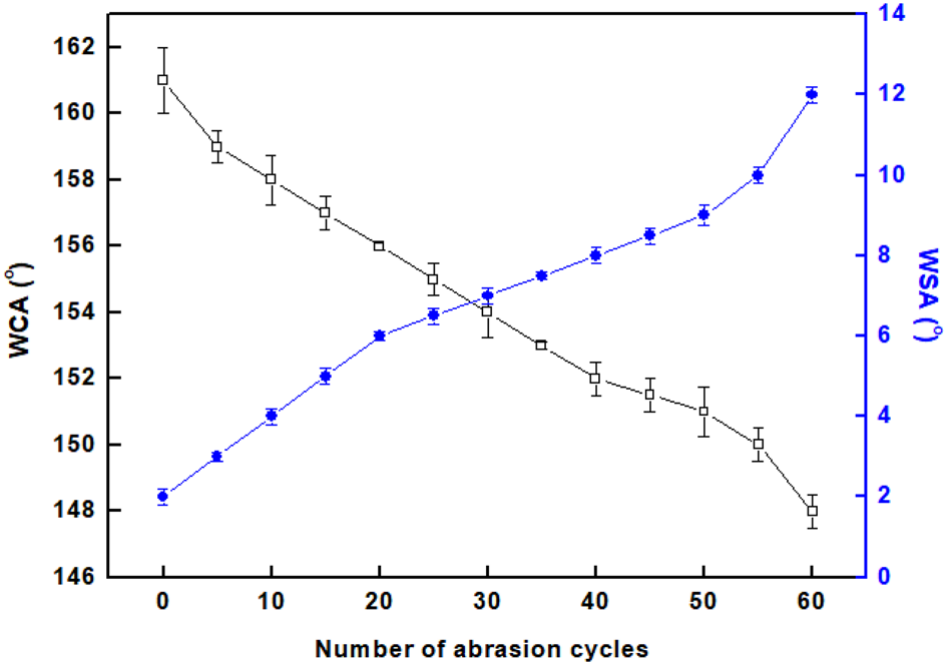
Variation of WCAs and WSAs with the abrasion cycles.

Poor mechanical abrasion of produced superhydrophobic coatings is viewed as a key problem in industrial applications. It has been shown that enhancing superhydrophobic coatings' abrasion resistance is essential for their industrial uses. The WCAs and WSAs after each five abrasion cycles are measured to demonstrate the variation in wettability, as shown in Fig. 11. The plots show that as the abrasion cycles increased, the WCAs reduced and the WSAs increased. The prepared superhydrophobic textile fabric maintains its superhydrophobicity until 55 abrasion cycles, where the WCA is greater than 150^o^, and the WSA is lower than 10°. The prepared superhydrophobic textile fabric has mechanical stability superior to several previously recorded values^94,100^.

### Chemical stability and Flux rate

**Figure 12:**
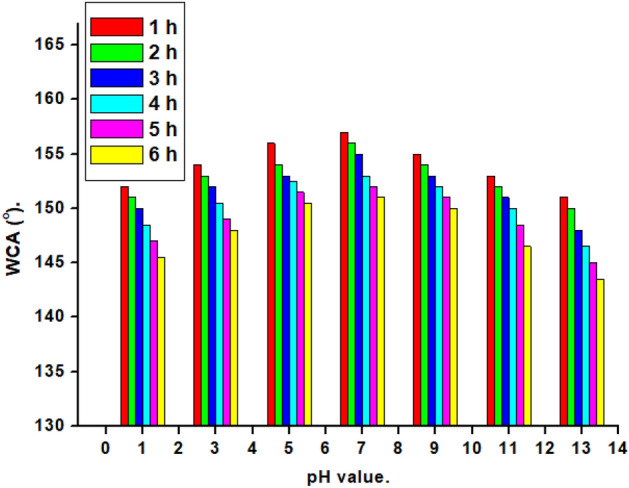
Influence of pH values on the modified textile fabric's water contact angle at different immersion times.

The stability of superhydrophobic surfaces is a critical issue for their practical uses. To establish the chemical stability of the created superhydrophobic textile fabric, the effect of acid and alkali solutions on the water contact angle was investigated. The prepared textile fabric was immersed in aqueous solutions of pH from 1 to 13 for different immersion times (1–6 h), and the WCA was estimated each one hour. The relation between the pH and WCA of the prepared superhydrophobic textile fabric for different immersion times is shown in Fig. 12. At pH 5–9, the WCA values are greater than 150°, showing that the modified textile fabric is still superhydrophobic. While the created superhydrophobic textile fabric loses its superhydrophobicity before 6 h of immersion in an extremely acidic and basic environment. The superhydrophobic nature is primarily responsible for effective oil/water separation capabilities, although most reported superhydrophobic coatings are chemically unstable. Furthermore, the chemical stability of the produced textile fabric (wt/wt) is superior to that of previously reported absorbents such as textile fabric modified with polyvinyl alcohol (WCA = 149°)^95^. Textile fabric modified with a fluoropolymer (WCA = 140°)^96^. Textile fabric grafted by silica aerogel and polydimethylsiloxane (WCA = 140°)^101^.

Due to its porosity and the rough features of the modified textile fabric, it is easy to form oil channels, which contribute to the high oil flux. The Flux of modified textile fabric was determined using Eq. (3). The modified textile fabric had a high oil flux rate. The Flux values for n-hexane, petroleum ether, silicone oil and n-hexane in water emulsion equal 15,700, 15,500, 15,400 and 8040 L m^−2^ h^−1^, respectively. The Flux stability of the modified textile fabric in the oil/water separation process was determined by measuring it after 10 cycles of separation processes. The Flux value for n-hexane, petroleum ether, silicone oil and n-hexane in water emulsion after 10 cycles of separation processes equal 14,900, 14,700, 14,500 and 7650 L m^−2^ h^−1^, respectively. It is obvious that the Flux is slightly decreased, reflecting the high Flux stability of the modified textile fabric.

The viscosities of various organic solvents are responsible for the variance in flux levels; Flux is inversely proportional to the viscosity of the liquid^102^. As a result, the created textile fabric can be utilized to effectively and quickly separate oil and water. Additionally, in comparison, these Flux rates are superior to those of reported absorbents, such as thiol-poly dopamine coated-textile (4500 L m^−2^ h^−1^)^100^. Textile fabric modified with lauric acid-TiO_2_ composites and Fe_3_O_4_ nanoparticles (7400–11,000 L m^−2^ h^−1^)^71^.

## Conclusion

A spray-coating technique was employed to achieve cost-effective and environmentally friendly superhydrophobic textile fabric for effective oil–water mixture and emulsion separation. The pristine textile fabric was turned into a superhydrophobic one by improving the surface roughness using Cu-Asp MOF and lowering the surface energy using stearic acid. The superhydrophobic textile fabric exhibits a high contact angle (158^o^ ± 1.3°), reflecting excellent superhydrophobic characteristics, and has a high absorption capacity of 85.4, 106.59, and 159.712 g/g for n-hexane, petroleum ether, and silicone oil. The superhydrophobic textile fabric has a good separation efficiency (95–99.4%) for the various oil/water mixes under investigation. The prepared modified textile fabric was successfully used for a Tween 80-stabilized n-hexane in water emulsion separation and has a high separation efficiency (90.1%). The prepared superhydrophobic textile fabric has high mechanical stability where it maintains its superhydrophobicity until 55 abrasion cycles. The prepared textile fabric exhibits high chemical stability, whereas it retains superhydrophobicity after immersion in aqueous solutions of pH from 1 to 13 for one hour. The created textile fabric has a high Flux rate (15,400–15,700 L m^−2^ h^−1^) for the different oil/water mixtures under study and a Flux rate of 8040 L m^−2^ h^−1^ for the n-hexane in water emulsion, so the prepared superhydrophobic textile fabric exhibits a unique application potential to effectively and quickly separate oil–water mixture and emulsion.

## Supplementary Information

Supplementary Video 1.

Supplementary Video 2.

Supplementary Video 3.

## Data Availability

The datasets used and/or analyzed during the current study available from the corresponding author on reasonable request.
